# Dynamic causal modeling of the working memory system of aneurysmal subarachnoid hemorrhage patients: Searching for targets for cortical intervention

**DOI:** 10.1002/brb3.2307

**Published:** 2021-09-14

**Authors:** Jie Sun, Nan Zhao, Jun Liu, Ze‐yi Wang, Ping Su, Jun‐yan Li

**Affiliations:** ^1^ Department of Neurosurgery The first Hospital of Kunming Kunming China; ^2^ Center of Cerebrovascular Disease Treatment Technology Kunming China

**Keywords:** dynamic causal modeling, independent component analysis, subarachnoid hemorrhage, working memory loss

## Abstract

**Introduction:**

Aneurysmal subarachnoid hemorrhage (aSAH), caused by rupture of an intracranial aneurysm and bleeding into the subarachnoid space, is a life‐threatening cerebrovascular disease. Because of improvements in clinical interventions, the mortality rate of aSAH is gradually decreasing. Thus, many survivors recover from aSAH but still have sequelae. Working memory (WM) deficit is one of the most common and severe sequelae after aSAH. Interestingly, the severity of WM deficit is not identical to the extent or localization of brain damage, which implies an underlying mechanism of WM deficit other than direct hemorrhagic brain damage. Previous studies have revealed altered neural activity of several brain regions during stimulus tasks. However, the behaviors and functional organization of these corresponding areas in the resting state remain unclear. Insights into the organization of the WM network could reveal novel information about the mechanism of WM deficits, which will be of great value in developing new therapeutic strategies.

**Methods:**

In this study, we recruited 50 aSAH patients consisting of survivors with either impaired or intact WM (two groups). Independent component analysis was performed on resting state data to extract the WM network. Dynamic causal modeling was then performed to assess the intrinsic coupling between key regions of the WM network. A model describing the neural activity and functional organization of the WM network was established, although some connections were not consistent in the resting state.

**Results:**

We found that effective connectivity of the precuneus (PCUN)‐middle temporal gyrus (MTG), MTG‐PCUN, and middle frontal gyrus‐inferior parietal lobule was significantly decreased in the impaired WM group, which suggests a vital and central role of affected regions or connections and provides new targets for brain stimulation.

**Conclusions:**

The results of this study may contribute to new therapeutic or rehabilitation strategies for aSAH patients with WM deficits.

## Graphical abstract

1. Our study suggests a vital and central role of affected regions or connections and provides new targets for brain stimulus. 2. We hope new therapeutic strategy or rehabilitation for aneurysmal subarachnoid hemorrhage patients with working memory deficit could benefit from this study.

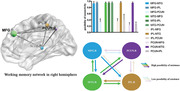

## 1 INTRODUCTION

Aneurysmal subarachnoid hemorrhage (aSAH) is an acute and life‐threatening cerebrovascular disease caused by intracranial aneurysm rupture and bleeding into the subarachnoid space (Rosengart et al., 2007). The mortality rate of aSAH is approximately 40%−50%, and most survivors suffer from severe neurological and cognitive impairment, which reduces their quality of life (Feigin et al., 2005). A previous study showed that SAH patients suffer from severe working memory (WM) deficits (Haug et al., 2007). Additionally, an animal study reported that SAH in rats is associated with significantly increased escape latency and swimming distance compared with controls in the Morris water maze (Takata et al., 2008).

Surprisingly, patients with similar WM loss do not show identical patterns of brain damage (Al‐Khindi et al., 2010; Mayer et al., 2002). Patients do not show simple sequelae corresponding to brain damage in specific regions, and the complicated process of dysfunction requires clarification. With the increasing prevalence of “high functioning” survivors, the etiology or mechanism of WM deficit is becoming a vital issue (Sheldon et al., 2013). The specific pathological alterations associated with WM deficit remain unclear. The first step is to describe the change in neural activity in aSAH patients with WM deficit. Then, the specific alterations can be located and analyzed, and potential targets for brain stimulation can be determined.

A previous study of task‐dependent activity revealed subcortical disconnection and subsequent decreased efficiency in neural processing in patients with WM deficit (Tariq et al., 2010). Memory does not consist of synaptic plasticity in a single neuron or cluster; it is encoded and represented by networks of neurons (Neves et al., 2008). Therefore, WM deficit may not involve impairment of an isolated brain region. Instead, abnormalities in complex networks should be explored. We suggest that insights into the organization of the WM network could reveal novel information about the mechanism of memory loss and may contribute to the development of new therapies for aSAH patients with WM deficit.

In the present study, we extracted the WM network using independent component analysis (ICA). Then, we performed dynamic causal modeling (DCM) analysis to calculate the effective connectivity between each pair of nodes in this network. Using an analysis of intrinsic coupling in the WM network, we aim to describe the functional organizational features of aSAH patients with memory loss. The results of this study could contribute to a preliminary understanding of the mechanism of WM deficit.

## 2 METHODS AND MATERIALS

### 2.1 Participants

A total of 50 aSAH patients who were treated and followed up for 5 years in our center were subjected to neuropsychological assessments and magnetic resonance (MR) scans, including 25 patients with intact WM and another 25 with WM deficits. Part B of the Trail Making Test was used to evaluate WM. The demographic characteristics and clinical status of the participants at the initial admission were retrospectively obtained and compared. The physical condition upon admission was evaluated according to the World Federation of Neurosurgical Societies grading scale, and the severity of aSAH was classified by head computed tomography (CT) using the Fisher grade (assessed by a neurologist and a radiologist). Medical treatment including nimodipine, antifibrinolytics, and antiepileptic drugs was applied based on the presence of complications of aSAH. Definitive treatment including clipping or coiling was performed within 48 h after the initial admission.

The inclusion criteria of this study were as follows: (1) patients older than 18 years, (2) SAH confirmed by CT scan, (3) aneurysm diagnosed by digital subtraction angiography, (4) hospital admission within 3 days of onset, and (5) Chinese speaking. The exclusion criteria were as follows: (1) patients with other diseases that may result in SAH, such as trauma and arteriovenous malformation; (2) surgical or interventional treatment before enrollment; and (3) declined to provide informed consent during follow‐up. This study was approved by the ethics committee of our hospital, and all the participants provided written informed consent.

Patients who survived the hemorrhagic attack spent circa 20 days in the neurology and neurosurgery department and another 60 days in a rehabilitation center. Neuroimaging and other tests were performed after discharge and initial rehabilitation, which was usually 75−90 days after admission to our center. A washout period of at least 30 days was used to eliminate the effect of previous medication.

### 2.2 Neuroimaging acquisition

Resting‐state functional magnetic resonance imaging (fMRI) data were obtained with a single‐shot gradient‐recalled echo planar imaging (EPI) sequence with the following parameters: interleaved scanning order, slice number = 49, matrix size = 64 × 64, field of view = 192 × 192 mm, repetition time = 2500 ms, flip angle = 89°, slice thickness = 3.0 mm, gap = 0 (voxel size 3.0 × 3.0 × 3.0 mm^3^), and number of acquisitions = 200.

An MRI‐safe clipping method was used to treat the enrolled patients (Khursheed, 2011). An advanced 3T MR scanner can successfully image brain tissue around implanted titanium aneurysm clips at different spatial ranges depending on the sequence type. The patients enrolled in this study had lesions distant from the regions of interest on fMRI analysis. Most of the clips had limited artifacts 2 mm outside of the boarder. In addition, construction of a spatial confidence boundary of signal integrity, represented numerically by an artifact mask volume, was performed. It was possible to quantify the degree of uncertainty across patients regarding whether the signal in a particular region of the brain was completely or partially affected by clip artifacts.

### 2.3 Data preprocessing

The Statistical Parametric Mapping toolbox (https://www.fil.ion.ucl.ac.uk/spm/) was used for data preprocessing. The functional data were first adjusted with a slicing time procedure to account for differences in acquisition times. Then, a head movement correction procedure was performed. Subjects with translational or rotational motion that exceeded 2.5 mm or 2.5° were excluded. Then, one mean volume was extracted and used as the reference image for realignment. Spatial normalization of the functional images was performed via a standard EPI template. Covariates including head motion parameters, white matter, and the cerebral spinal fluid blood‐oxygen level‐dependent signal were regressed from functional data. Finally, spatial smoothing with a 6‐mm full width at half maximum Gaussian kernel was performed for denoising.

### 2.4 Independent component analysis

**FIGURE 1:**
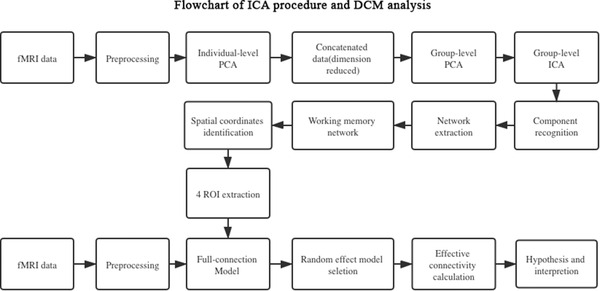
Main processes of independent component analysis (ICA) and dynamic causal modeling analysis. Preprocessing of functional magnetic resonance imaging data included slice timing, realignment, normalization, and smoothing. ICA was performed with concatenated images of both groups. The full connectivity model was established with four nodes, and then random effect model selection was used to screen possible candidates

The Group ICA toolbox was used to analyze the fMRI data. Generally, a group ICA with a concatenation approach plus back reconstruction is used to analyze multiple subjects (Calhoun et al., 2001). Dimension reduction was first performed on the functional data. Then, the number of independent components of subjects was decreased to 60. Next, temporal concatenation was performed to connect images. All images were reduced to 30 components at the group level using the expectation maximization algorithm. Additionally, 100 repetitions of the infomax algorithm were performed to increase robustness. After the aggregated spatial maps were estimated, the back reconstruction approach was used to extract subject‐specific spatial patterns and time courses. The spatial weight maps were thresholded with a significance level of *p* < .05. After the spatial patterns of certain components were revealed, we manually identified nine sub‐networks including the WM network. Components that showed the spatial pattern of the WM network were binarized and transformed into a mask to extract the time series of the WM network from the original data. In this procedure, the spatial coordinates of several peak points that represent the highest probability of belonging to the current component were determined (Figure 1).

### 2.5 Dynamic causal modeling

Based on the current understanding of brain activity in the resting state, classical DCM with fixed effect Bayesian model selection cannot process low‐frequency fluctuation because it assumes that brain activity in each region is only triggered by external stimulation, which fails to explain the resting state of the brain and its effective connectivity. Therefore, DCM with network discovery was used to explore all of the possible models after preprocessing and ICA (Friston et al., 2011). We established a full connectivity model and estimated the probability of existence for every potential edge. Edges with a low probability of existence (less than 50%) were excluded from the model. Only intrinsic coupling, which is represented as matrix A in the neural state model, was calculated (Friston et al., 2003). Because no stimulus task is performed in the resting state, modulation of connectivity (Matrix B) and direct input (Matrix C) were not priorities.

### 2.6 Statistical analysis

For clinical assessments, quantitative variables are shown as means ± SD, and categorical variables are expressed as proportions. A two‐sample *t* test was used for comparisons of quantitative variables between two groups if normality tests and homogeneity of variance were satisfied. Otherwise, a Wilcoxon test was used. Statistical significance was set as a two‐tailed *p* value less than 0.05.

For ICA, a one‐sample *t* test was used to compare the spatial weight maps to reveal the pattern of each component. The initial significance level was set to *p* < .05. However, this level was flexible. The significance level could be adjusted if a better spatial pattern of components was revealed.

In the intrinsic coupling analysis, an unpaired *t* test and Welch correction were performed and the significance level was set to *p* < .05.

## 3 RESULTS

### 3.1 Participants

The demographic and clinical factors of the two groups upon initial hospital admission did not significantly differ (Table 1). As expected, the WM status of the two groups significantly differed at the follow‐up evaluation (*p* < .001).

**TABLE 1:** Demographic characteristics of the two groups

	Memory intact group (*n* = 25)	Memory defected group (*n* = 25)	Statistic
Age at admission (years)	52.1 ± 12.0	58.5 ± 9.6	0.076
Gender at admission (%)	12 (48.0)	11 (44.0)	>0.05
WFNS grade IV‐V at admission (%)	5 (20.0)	4 (16.0)	N/A
Modified Fisher grade at admission	1.6 ± 1.3	2.1 ± 1.4	0.261
Follow‐up TMT‐B (s)	90.7 ± 15.9	160.3 ± 52.9	<0.001

Abbreviations: TMT‐B, Trail Making Test Part B; WFNS, World Federation Neurological Surgeons.

### 3.2 ICA results

**FIGURE 2:**
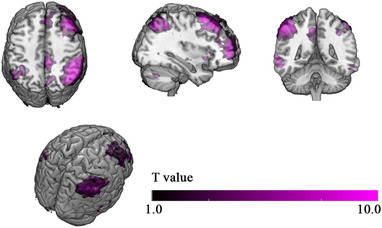
Spatial pattern of the working memory network extracted by independent component analysis (ICA). A one‐sample *t* test was used to threshold the spatial maps after ICA. The value of each voxel represents the probability that this voxel belongs to the current component. The largest significant clusters included the right inferior parietal lobule, right middle frontal gyrus, right middle temporal gyrus, and right precuneus. The statistical threshold was set at *p* < .05 with no correction

After ICA, the spatial pattern map of the WM network was extracted (Table 2, Figure 2). The coordinates of the peak points of the four largest clusters, including the right inferior parietal lobule (IPL, [54, −39, 51]), right middle frontal gyrus (MFG, [42, 51, 6]), right middle temporal gyrus (MTG, [66, −48, 3]), and right precuneus (PCUN, [9, −45, 45]) were determined. These peak points were regarded as nodes in the full connection model in the subsequent DCM analysis.

**TABLE 2:** Significance clusters in the working memory network extracted by independent component analysis (results of a one‐sample *t* test)

	MNI coordination				
Region	x	y	z	Voxels	Peak intensity
IPL.R	54	−39	51	902	9.52
MFG.R	42	51	6	1401	9.39
MTG.R	66	−48	3	202	6.93
PCUN.R	9	−45	45	223	5.16
MFG.L	−39	45	9	35	6.23
MCC.L	9	33	39	176	5.87

*Note*: x, y, z, coordinates of primary peak locations in the Montreal Neurological Institute (MNI) space.

Abbreviations: IPL.R, right inferior parietal lobule; MCC.L, left medial cingulate cortex; MFG.R, right medial frontal gyrus; MFG.L, left medial frontal gyrus; MTG.R, right medial temporal gyrus; PCUN.R, right precuneus.

### 3.3 DCM analysis results

**FIGURE 3:**
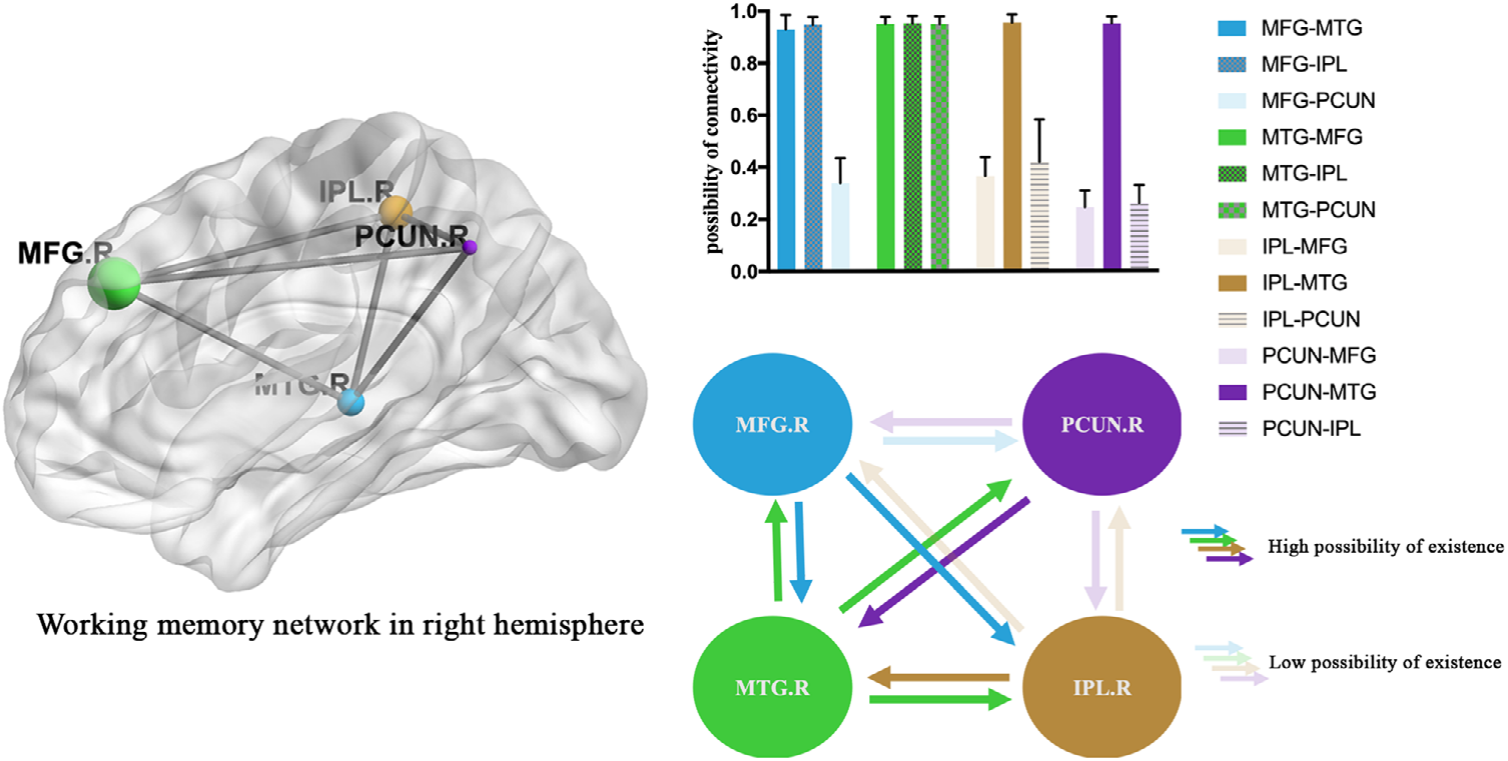
Estimation of the probability of existence of edges according to dynamic causal modeling analysis. After selection of the random effect model, connections between the right middle frontal gyrus (MFG.R)‐precuneus (PCUN), inferior parietal lobule (IPL)‐MFG, IPL‐PCUN, PCUN‐MFG, and PCUN‐IPL did not show a high probability of existence (less than 50%). They are shown as partially transparent in the figure. Connections with a high probability of existence are shown with saturated colors

In the established model, connections between the MFG‐MTG, MFG‐IPL, MTG‐MFG, MTG‐IPL, MTG‐PCUN, IPL‐MTG, and PCUN‐MTG showed a high probability of existence (Figure 3). The remaining edges in the model did not show a high probability of existence (less than 50%) and were not included in the subsequent analysis.

**FIGURE 4:**
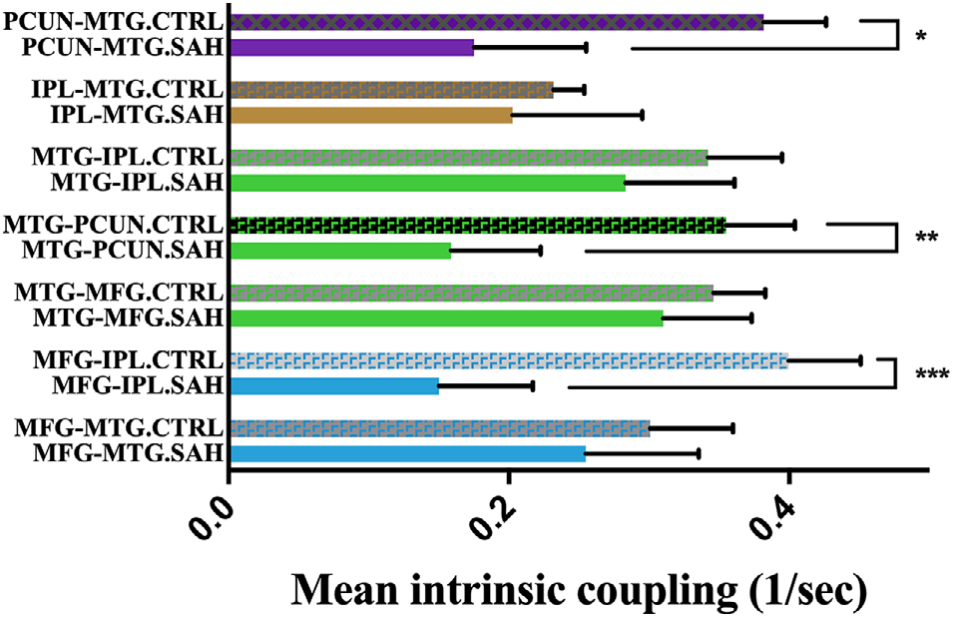
Statistical analysis of intrinsic coupling in the two groups. connections of the precuneus (PCUN)‐middle temporal gyrus (MTG) (*), MTG‐PCUN (**), and middle frontal gyrus (MFG)‐inferior parietal lobule (IPL) (***) were significantly decreased in aneurysmal subarachnoid hemorrhage patients with memory loss. An unpaired *t* test and Welch correction were performed for comparisons. The significance level was set at *p* < .05

We compared the intrinsic coupling between the WM deficit group and the intact group and found that the effective connectivity of the PCUN‐MTG, MTG‐PCUN, and MFG‐IPL was significantly decreased (*p* < .01) in the WM deficit group. The remaining comparisons showed no significant difference between groups (Figure 4).

## 4 DISCUSSION

Our study focused on the functional organization of the WM network in aSAH patients with WM deficits after long‐term follow‐up to provide novel information on regional intrinsic coupling alterations for the first time. The main findings were as follows: (a) four key regions are present in the WM network of aSAH patients, including the MFG, MTG, IPL, and PCUN; (b) some connections between these regions are not as consistent as the others in the resting state; (c) the WM deficit group showed significant decreases in intrinsic coupling of the PCUN‐MTG, MTG‐PCUN, and MFG‐IPL. Overall, the analysis revealed a significant alteration in the WM network, and we hypothesize that this is a possible explanation for WM deficits in some aSAH patients.

In the ICA, images of both groups were concatenated to perform group‐level dimensional reduction. Although the two groups may exhibit differences in brain activity, we reasoned that the extracted spatial pattern of components should be sufficiently similar in both groups for statistical comparison. Therefore, the spatial maps of components representing the WM network are similar to those of previous human studies (Damoiseaux et al., 2008, 2006). In addition, our results do not include other WM network patterns in which most of the significant voxels are localized in the left hemisphere, which may be due to the limited sample size of our study. The components of the WM network in the left hemisphere failed to achieve the threshold of within‐group significance.

In the analysis of the full connectivity model, some connections with a low probability of existence in the resting state were screened out. The most interesting and valuable findings were related to the bidirectional intrinsic connections of the PCUN and MTG. In fact, the only pathways to and from the PCUN in the WM network were affected in patients with impaired WM because the other connections were not consistently present in the resting state. The PCUN is located in the superior parietal lobule on the medial surface of each brain hemisphere. Previous fMRI studies reported its computational connections with the caudate nucleus, parahippocampal area, and superior temporal area (Zhang & Li, 2012). However, no anatomical connection with the inferior parietal area, prefrontal area, or primary motor area has been reported. We suggest that this lack of anatomical connection could explain the results of our existence analysis.

The PCUN is highly involved in memory tasks, especially those requiring spatial details (Wallentin et al., 2006). In complicated tasks such as recollecting a memory, the PCUN functions with the hippocampus to discern contextual information. In this manner, the PCUN is involved in diverse cognition processes including WM and episodic memory retrieval (Cavanna & Trimble, 2006; Margulies et al., 2009). However, the MTG is also a critical structure for long‐term memory. The MTG, along with the surrounding hippocampal regions, is believed to be involved in encoding declarative long‐term memory (Squire et al., 2004). As demonstrated in our study, the MFG, MTG, and IPL are densely connected, and the PCUN is connected bilaterally only with the MTG. This suggests a vital and central role of the MTG in patients with WM loss. The MTG seems to be the hub region in the local WM network. Our results indicated that the intrinsic coupling of both the MTG‐PCUN and PCUN‐MTG was significantly decreased in the WM deficit group. These findings clearly indicate that the neural pathway between the MTG and PCUN is a vital region for WM deficits, but the specific pathological process is not clear. This result provides novel insights for therapy involving stimulation of the MTG, which may have potential benefit for rehabilitation of patients with aSAH.

The other noteworthy finding of our study is the unidirectional connection of the MFG‐IPL. The lack of an IPL‐MFG connection implies a lower rank of the IPL in the regional WM network, as the IPL only receives input from the MFG and does not sent output. Emerging evidence has linked processing in the IPL to declarative memory. Bilateral damage to this brain region does not cause amnesia; however, the strength of memory is diminished, details of complex events become more difficult to retrieve, and subjective confidence in memory decreases (Berryhill et al., 2007; Hower et al., 2014). The MFG consists of the precentral area and prefrontal area. A previous study suggested that the prefrontal area mostly performs short‐term maintenance of information. It does not focus on the manipulation or monitoring of such information or on the use of that information for decisions (Lebedev et al., 2004). This implies that the MFG serves as a “memory flash drive” and sends information to other functional modules. According to our results, the IPL receives such information and acts as a complex memory processor in the WM network in the resting state. Considering that memory loss patients show a significant decrease in the MFG‐IPL connection, we reasoned that the MFG, which is regarded as a “memory flash drive,” is one of the most important areas related to WM deficit. Along with the previous analysis, we provide several potential targets for cortical interventions such as transcranial magnetic stimulation. The results of this fMRI study may contribute to new therapeutic strategies for aSAH patients with WM deficits.

## 5 CONCLUSION

WM deficit is a common sequela of aSAH patients that does not strictly correspond to the anatomical damage to the brain. Our study provides insight into the functional organization of the WM network using DCM. The results show a significant decrease in intrinsic coupling of the MFG‐IPL, MTG‐PCUN, and PCUN‐MFG, providing novel information and a theoretical foundation for new therapeutic brain stimulation strategies.

## FUNDING INFORMATION

Science and Technology Research Project of Xishan District(Num: Xi‐25,2020), Kunming Province.

### PEER REVIEW

The peer review history for this article is available at https://publons.com/publon/10.1002/brb3.2307.

## Data Availability

All of the data are available upon reasonable request from the corresponding authors.
